# A Qualitative Analysis of User Experiences With a Self-Tracker for Activity, Sleep, and Diet

**DOI:** 10.2196/ijmr.2878

**Published:** 2014-03-04

**Authors:** Jeongeun Kim

**Affiliations:** ^1^Seoul National UniversitySeoulKorea, Republic Of

**Keywords:** self-tracker, quantified-self, health consumer, qualitative research

## Abstract

**Background:**

The recent increase in chronic diseases and an aging population warrant the necessity of health self-management. As small electronic devices that track one’s activity, sleep, and diet, called self-trackers, are being widely distributed, it is prudent to investigate the user experience and the effectiveness of these devices, and use the information toward engineering better devices that would result in increased efficiency and usability.

**Objective:**

The aim of this study was to abstract the constructs that constitute the user experiences of the self-tracker for activity, sleep, and diet. Additionally, we aimed to develop and verify the Health Information Technology Acceptance Model-II (HITAM-II) through a qualitative data analysis approach.

**Methods:**

The study group consisted of 18 female college students who participated in an in-depth interview after completing a 3-month study of utilizing a self-tracker designed to monitor activity, sleep, and diet. The steps followed in the analysis were: (1) extraction of constructs from theoretical frameworks, (2) extraction of constructs from interview data using a qualitative methodology, and (3) abstraction of constructs and modeling of the HITAM-II.

**Results:**

The constructs that constitute the HITAM-II are information technology factors, personal factors, social factors, attitude, behavioral intention, and behavior. These constructs are further divided into subconstructs to additionally support the HITAM-II.

**Conclusions:**

The HITAM-II was found to successfully describe the health consumer’s attitude, behavioral intention, and behavior from another perspective. The result serves as the basis for a unique understanding of the user experiences of HIT.

## Introduction

### Self-Tracking Benefits

The philosophy behind the Quantified Self movement is best described by the phrase “You are your data;” it aims to improve various aspects of life and health through recording and reviewing daily activities and biometrics. It is noted that people seeking greater self-knowledge, when using numbers on this quest to understand themselves, experienced a positive and accelerated path towards their desired goals [1]. Appropriately, many new online communities are being founded where people with shared interest in self-tracking can have active discussions and share their knowledge with others. CureTogether is one of the prime examples where patients can share data and self-report symptoms, treatments, and triggers for over 300 conditions. The quantitative data at CureTogether enables decision support and hypothesis generation [2]. Another example of a similar health community is PatientsLikeMe, a health social network service. Within it, patients suffering from a motor neuron disease (namely, amyotrophic lateral sclerosis) curated a huge database on the outcomes of lithium carbonate treatment. Based on this database, the patients found that within the first 12 months lithium had no effect on the progression of their diseases. This was a powerful case of data curated through patients on the Internet serving as a critical tool for accelerating clinical discovery and evaluating the effectiveness of drugs already in use [3]. Ultimately, self-tracking not only benefits the individuals who are actively collecting the data in diagnosis and finding the best possible treatments, but also the sharing of such data can be integrated into traditional pharmaceutical and medical research that may result in wider impacts to the health community. Therefore, it is imperative to formally investigate these new devices so that significant improvements in the health care industry can be made.

### Medicine 2.0 and Web 2.0

On the other hand, Medicine 2.0 emerged in the market nearly simultaneously and provides Web-based services for health care consumers, caregivers, patients, health professionals, and biomedical researchers. Those using Web 2.0 technologies and/or semantic Web and virtual-reality tools are able to facilitate social networking, participation, apomediation, collaboration, and openness within and between user groups [4]. However, for Medicine 2.0 to deliver on its promises of collaboration, participation, and social network applications, an infrastructure that allows accurate measurement, systematic classification, and continuous management of the health record is required.

Unless something can be measured, it cannot be improved. So we are on a quest to collect as many personal tools that will assist us in quantifiable measurement of ourselves [2].

Kevin Kelly of the Quantified Self blog

This suggests that health consumers need a set of easy-to-use tools that keep them motivated to track themselves, as well as tools that make sense of the tracked data and provide actionable lessons; and it is very likely that self-trackers will be able to address these various issues [1].

### Self-Trackers and Smartphones

The use of self-trackers is increasing rapidly. The rapid advances in smartphone technology have resulted in a strange new concept of an “external brain” that expands the capacity of the biological counterpart. The modern population now relies on their smartphones and mobile computers with an emotional and cognitive fervor that was never before seen. By possessing a second self, people communicate, think, and act through these devices. This phenomenon is now accompanied by the so-called “digital dementia,” where people store their knowledge in external devices and no longer “remember” them in the traditional sense. Ray Kurzweil, the author of “The Singularity is Near,” has said that smartphones are “brain extenders,” and that even without being physically embedded into one’s body, people are already deeply reliant on it [5]. Aside from smartphones, new self-tracking devices like Fitbit [6], Flex, Aria, and BodyMedia are available to track and record people’s activity, sleep, and diet, as well as providing supplementary services like Web-based data management tools, data sync, and storage.

### People Accepting Health Information Technology

There are many factors contributing to people using health information technology and self-trackers to manage their health. Several studies have addressed factors like technology acceptance or innovation adoption, that describe the acceptance, adoption, and utilization of information technology, and two major theories have resulted from these studies. These theories are the Technology Acceptance Model (TAM) by Davis [7] and the Diffusion of Innovation by Rogers [8]. Building upon these theories, Kim and Park developed a model that describes the process of people’s acceptance and use of Health Information Technology (HIT) for health management called the Health Information TAM (HITAM).

In 2011, IBM conducted research on health and wellness devices to identify the requirements for health devices that would enable wide distribution and increased benefits. Using an interviewing methodology, the research cast a wide net on current device users, caregivers, medical device makers, and consumer electronics companies alike. There is no precedence of investigations into the user’s perspectives and experiences using in-depth data analysis [9]. This study aims to address this gap by using a prime example of HIT, self-trackers, and individually interviewing the device users. The study proposes to process and analyze the user experience.

The questions for this research are: (1) What are the experiences of self-tracker users with activity, sleep, and diet?; (2) how to describe those experiences using relevant constructs and a model of HITAM; and (3) As a model describing health information technology acceptance, what are the differences between the results of construct modeling through quantitative analysis and construct extraction through qualitative analysis?

To address the research questions: (1) the first aim of this study is to abstract the constructs that constitute the user experiences of the self-tracker for activity, sleep, and diet; and (2) the second aim of this study is to develop and verify the HIT Acceptance Model-II (HITAM-II) by the qualitative data analysis approach.

The findings of the research will provide unique perspectives on various aspects of user attitude, intention, and actual usage behavior when accepting HIT.

## Methods

### Participant Age Range for the Study

Even though the aged population and the patients who suffer from chronic diseases would most benefit from utilizing self-tracking and monitoring of their health status, this population has not yet adopted it. Because of that reason, to survey the experiences of self-tracker users, participation requests were sent out to female university students 20-29 years of age. They were chosen because they possess a high level of interest in diet and health related issues, and a negligible amount of resistance towards experimenting with new technology. Since the methodology uses quasi-experimental research, the participation was strictly volunteer based, and the Institutional Review Board of the educational institute’s approval was secured prior to the start of the survey.

### Participant Recruitment

Initially, the authors approached and verbally recruited the participants. Snowball sampling was then used to identify additional participants until the desired number was recruited. They were given the self-tracker named Fitbit, and the self-tracking survey began in December 2010 and ended in March 2011, with a total of 44 participants. The participants were asked to attend an hour long orientation session. The session consisted of presentations on the research objectives and methodology, instructions on how to use the self-tracker device, consent forms completion, registration of user accounts on the self-tracker website, and instructions on how to contact the survey administrators in case of questions or concerns. The participants were asked to track and monitor their activity, sleep, and diet for a minimum of three months.

### Group Interview

At the end of the survey, 18 students consented to participate in a group interview designed to gather various opinions and impressions regarding the self-tracker. The interview was based on the focus group interview format where questions are posed to the entire group and people are encouraged to answer freely in a conversational setting. The interview lasted approximately two hours and the questions were predetermined in a semistructured questionnaire format (Table 1). However, the questionnaire was used in a flexible manner according to the flow of the interview. The entire proceeding was audio-recorded, which was transcribed into a text file afterwards.

**Figure 1:**
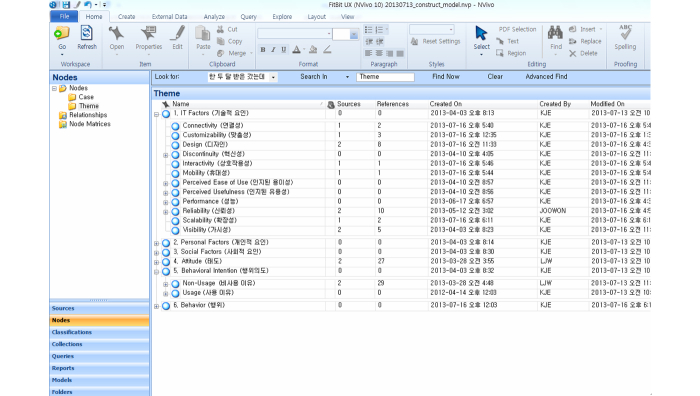
Screen capture of the constructs coding using NVivo qualitative analysis software.

**Figure 2:**
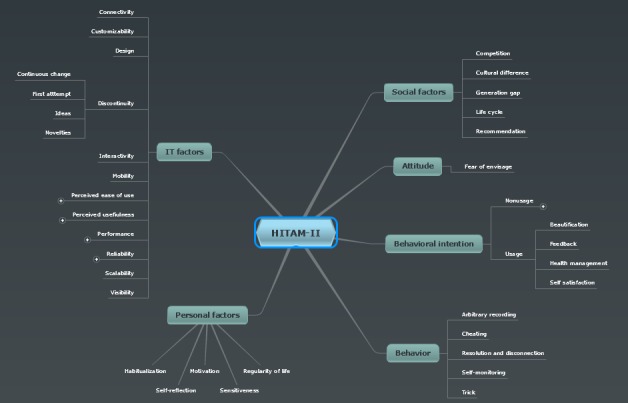
The first two out of three tiers of constructs in HITAM-II.

To validate the emergent constructs using the experimental data, a structured qualitative analysis was conducted. The procedure of the analysis is as follows: (1) Stage 1-extraction of constructs from theoretical frameworks-through literature survey, seven theories regarding the information technology acceptance, adoption, and user behavior were identified [7,8,10-14]. For systematic extraction of constructs from theoretical frameworks, the primary constructs of the seven theories were compiled (Table 2). Based on the analysis, we identified the constructs unique to each theory and the constructs common to all theories. These constructs were categorized into two levels-upper and lower levels-according to the scope. The upper level included Information technology (IT) factors, Personal factors, Social factors, Attitude, Behavioral intention, and Behavior, and the lower level included the rest of the constructs. (2) Stage 2-extraction of constructs from the interview data using qualitative methodology-for systematic analysis of the qualitative data, NVivo v10.0 was used to maximally extract the various constructs expressed by the participants’ languages (Figure 1 shows the constructs coding). The extraction of constructs workflow was performed iteratively, where the content of the data were analyzed repeatedly and investigated following the standard qualitative methodology. To visually represent the connectivity between the constructs, a mind map diagram was used (Figure 2 shows the mind map diagram). And finally, (3) Stage 3-abstraction of constructs and modeling of the HITAM-II-The union set of constructs identified through the literature survey in Stage 1 and the constructs extracted from qualitative analysis of the experimental data in Stage 2 were compared. Because the two stages were conducted independently of each other, the initial analysis yielded some disagreements over the various constructs, and settling the different tiers proved difficult. However, through in-depth comparative analysis of reconciling the constructs from both stages, a unified framework was established. In the framework, the constructs that were identified in Stage 1 that were not extracted in Stage 2 were eliminated, whereas newly identified constructs from Stage 2 that were not present in Stage 1 were added.

**Table 1:** Sessions and guiding questions of the focus group interview.

Session	Questions	Category
Intro	Simple “ice-breaking comments” and questions to make the participants understand the objectives of the research and ready for the interview.	
Transition	Have you ever heard about the cutting-edge information technology that could be used in health care and heath management? Have you ever used any health devices or smartphone applications other than the device used for this research?	
Main issue	What were the main benefits that you have had using the Fitbit device? Was it useful for health promotion in general, or diet in specific, for example?	Pros
	What was the most difficult aspect of using the device? –methods of the usage of the device itself –management and/or utilization of the device	Cons
	Do you have any good ideas to resolve these difficulties? What should be revised or added to make the device smarter to use?	Upgrade
	How was your experience of using the website of the device? Did you ever utilize the information uploaded for health promotion or diet? Did you have any difficulties in information management or utilization of the website?	Website
	Have you ever recommended the use of the device to your family, friends, and colleagues? Would you recommend the device to your family, friends, and colleagues? Who do you think would get the benefit of using the device from the perspective of consumer?	Utilization
	Are you willing to purchase the device for $99.95 and pay $49.99/year to purchase the premium membership of the device?	Value
	What is your overall experience with the device? Was it positive or negative? Please explain it in detail.	Evaluation
Wrap-up	Summary and revisit any issue if needed.	

**Table 2:** Constructs from relevant theories explaining the acceptance/adoption of information technology by users.

	Constructs emerged	HITAM [10]	TAM [7]	TAM 2 [11]	UTAUT^a^ [12]	Attributes of innovation [8]	Adoption of information technology innovation [13]	Innovation adoption model [14]
IT factors	HIT characteristics	HIT characteristics						
	Perceived usefulness	Perceived usefulness	Perceived usefulness	Perceived usefulness				
	Perceived ease of use	Perceived ease of use	Perceived ease of use	Perceived ease of use			Ease of use	
	Output quality			Output quality				
	Product performance							Product performance
	Performance expectancy				Performance expectancy			
	Customizability							Customizability
	Observability					Observability		Observability
	Result demonstrability			Result demonstrability			Result demonstrability	
	Visibility						Visibility	
	Communicability							Communicability
	Discontinuity							Discontinuity
	Category risk							Category risk
	Image			Image			Image	
	Job relevance			Job relevance				
	Complexity					Complexity		
	Complexity in use							Complexity in use
	Complexity in design							Complexity in design
	Trialability					Trialability	Trialability	Trialability
Personal factors	Demographic variables	Demographic variables			Gender, age			
	Subjective norm	Subjective norm		Subjective norm				
	HIT self-efficacy	HIT self-efficacy						
	Experience			Experience	Experience			
	Effort expectancy				Effort expectancy			
	Personal compatibility							Personal compatibility
Social factors	External variables		External variables					
	Social influence				Social influence			
	Facilitating conditions				Facilitating conditions			
	Compatibility					Compatibility	Compatibility	
	Social compatibility							Social compatibility
	Relative advantage					Relative advantage	Relative advantage	Relative advantage
	Social advantage							Social advantage
	Relative economic advantage							Relative economic advantage
Attitude		Attitude	Attitude					
Behavioral intention		Behavioral intention	Behavioral intention to use	Intention to use	Behavioral intention			
	Volition							Volition
	Voluntariness			Voluntariness	Voluntariness of use		Voluntariness	
Behavior		Behavior	Actual system use	Usage behavior	Use behavior			

^a^UTAUT: Unified Theory of Acceptance and Use of Technology

## Results

### The Theoretical Framework

The theoretical framework established is a version of the HITAM proposed by Kim and Park [10] with modifications based on the findings of the survey. Primarily, the factors that affect the experiences of the user in HIT devices, such as self-trackers, are categorized into IT, Personal, and Social factors. In addition, the unique aspects were presented in the Attitude, Behavioral intention, and Behavior categories.

### The Constructs and Subconstructs

**Figure 3:**
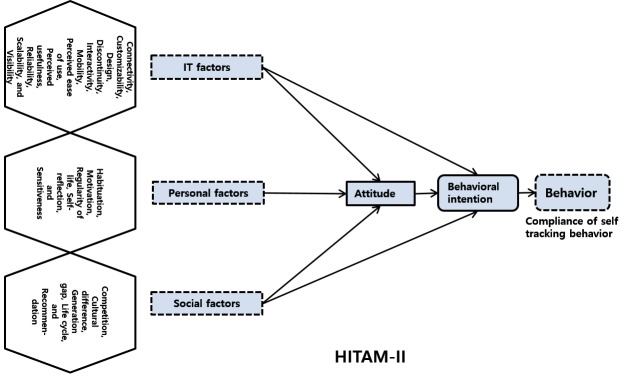
Theoretical framework of HITAM-II.

First, the IT factors yielded a total of 11 constructs–Connectivity, Customizability, Design, Discontinuity, Interactivity, Mobility, Perceived ease of use, Perceived usefulness, Reliability, Scalability, and Visibility (Figure 3 shows these constructs). Among them, the following constructs were further divided into subconstructs (shown in parenthesis)–Discontinuity (Continuous change, First attempt, Ideas, and Novelties), Perceived ease of use (Automation, Convenience, and Fun), Perceived usefulness (Effectiveness, Functional usefulness, and Health management), Performance (Guideline, Multipurpose, and Self-tracking), and Reliability (Inferior goods, and Operational error). Second, the Personal factors yielded a total of 5 constructs–Habituation, Motivation, Regularity of life, Self-reflection, and Sensitiveness. Third, the Social factors yielded a total of 5 constructs–Competition, Cultural difference, Generation gap, Life cycle, and Recommendation. Fourth, the Attitude aspect yielded a single construct, Fear of envisage, and the Behavioral intention aspect yielded two constructs Nonusage and Usage. These two constructs are divided into the following 13 (shown in parenthesis) and 4 subconstructs (shown in parenthesis)–Nonusage (Abandonment, Cost, Forgetfulness, Inconvenience, Language barrier, Life pattern change, Lost, Lost willpower, Low priority, Reliance, Seriousness, Sustainability, and Uselessness), and Usage (Beautification, Feedback, Health management, and Self-satisfaction). Finally, the Behavior aspect yielded five constructs, Arbitrary recording, Cheating, Resolution and disconnection, Self-monitoring, and Trick. The extracted constructs, their detailed structure, and each subconstruct accompanied by a representative transcription of the interview are summarized in a supplementary table (see Multimedia Appendix 1).

## Discussion

### Principal Findings

Our research employed qualitative data analysis methodology in order to complement the original efforts in developing the HITAM [10], as well as to supplement it with more in-depth analysis of user experience. The basis that formed the model was collected from the various information technology acceptance/adoption theories that share mutual objectives with this study and the constructs that compose the theoretical frameworks [7,8,11-14]. Our findings are unique from the previous approaches in that, instead of being the result of focusing on the description of technology acceptance/adoption, various health related factors were the main focal points of the study. Therefore, many nonoverlapping concepts were newly uncovered in this study, while some of the concepts previously identified did not emerge in our result. Additionally, the research confirmed the primary distinction between quantitative and qualitative analysis methodologies previously observed; as opposed to quantitative analysis methodologies where the majority of the survey variables are predetermined, qualitative analysis accepts new concepts that get uncovered throughout the analysis process. This highlighted the relatively construct-free analytical approach where a priori knowledge of the surveyor does not limit the outcome of the analysis.

### Quantitative and Qualitative Research Methodologies

In this study, we employed a hybrid approach called methodological triangulation, where both quantitative and qualitative research methodologies are used depending on the research objectives and various stages of the research. There are three advantages to using methodological triangulation. The first advantage is completeness-quantitative methods can further develop findings derived from qualitative research and vice versa. These methods complement each other, providing richness or detail that would be unavailable from using one method alone. The second advantage is abductive inspiration. In the cases of research where a phenomenon is poorly understood, interviews with participants can orient the investigators to the appropriate material. This can lead to hypotheses that can be verified through quantitative methods. Furthermore, qualitative investigation can also help organize quantitative data that has already been gathered or suggest new ways of approaching the phenomenon. The third and the most controversial advantage is confirmation. Qualitative methods can clarify apparently inconsistent findings found in quantitative results, even in its most modest form. More tendentiously, qualitative and quantitative results can sometimes support each other. Triangulation would thus yield a stronger result than either method could yield alone [15]. Therefore, one of the contributions of the study lies in implementing a relatively novel research methodology in approaching a research question where a previously unidentified phenomenon is investigated.

The rapid advance in computer and Internet technology in the 21st century caused nearly all manner of human and environmental aspects–including methods and philosophy in scientific research–to gravitate towards digitalization. The phrase “Big Data” did not even exist a decade ago, but is now an integral phrase in countless research disciplines, and a fundamental shift in statistical methodologies that handle “Big Data” is predicted. Furthermore, it is predicted that the research methodologies for collecting and interpreting human knowledge will become more diversified as well as refined.

### Conclusions

This study contributed to such a trend in research methodology by addressing the user experience of HIT adopters, where a number of questions arise at the interface of health and information technology. Strikingly, we identified various subtle changes in the user emotion and psyche caused by self-tracking, self-reflection, self-management, and data recording. Some interesting examples included falsifying their records (both intentionally and unintentionally), failing to meet self-set goals when users temporarily felt relieved from the constant “survey” by the self-tracker, and altering their daily behaviors in order to simplify the recording process. All of these user experience accounts—including psychological and behavioral changes—will provide invaluable insights into developing the next generation of HIT devices that will seamlessly integrate into daily human lives while tracking, monitoring, recording, transferring, and utilizing various health and biometric data. This study makes its major contribution in providing the basis of understanding of the three factors—IT, Personal, and Social—and the subsequent Attitude, Behavioral intention, and Behavior that affect the self-tracking behavior with the intention of health management.

## Multimedia Appendix 1

Constructs and statements of the HITAM-II.

## Abbreviations

HIT: Health Information Technology
HITAM: Health Information TAM
HITAM-II: Health Information Technology Acceptance Model-II
IT: Information technology
NRF: National Research Foundation of Korea
TAM: Technology Acceptance Model
