# Supplementary material for: A Qualitative Analysis of User Experiences With a Self-Tracker for Activity, Sleep, and Diet
Source: Interact J Med Res. 2014 Mar 4;3(1):e8. doi: 10.2196/ijmr.2878 (PMC3961803; doi:10.2196/ijmr.2878)
Supplement: Supplementary file 1 [file ijmr_v3i1e8_app1.pdf]

Category	Construct	Subconstruct	Statement
IT factors	Connectivity		I think if this was an app, it would be totally different. Like, make the device itself connect to a phone using a plug.. or bluetooth would be cool...
	Customizability		You know how the device is pre-set. How about making it so that you wear it for a week as a test, and the device tracks when you're active and when you're not, and then automatically start the sleep mode when it's near the person's sleep time.
	Design		This thing is so teeny. And if you don't see it, you forget it. Whether I'm busy or not, if it's right there I'd take it with me. But if I don't see it in the morning on my desk, I'll just forget about it. When I'm out the door, I'd be like, oh I forgot! I know it's small and portable, but looking for it is such a hassle, and remembering isn't easy.
	Discontinuity	Continuous change	I think it needs to change more frequently for me to remain interested. If it's all the same after awhile, I get used to it and lose interest. Maybe at an appropriate timing? Though, I don't know what that would be..
		First attempt	A smart person comes up with an idea, and then it gets made and comes into our lives through multiple steps. Sure some ideas make lots of money. They must go, oh this would totally catch on, but there are too many of those.
		Ideas	The problem with engineers is that they have these GREAT ideas, but there's also a huge gap between the actual implementation. What I mean is that there are endless number of new U-health devices and apps in development. But where do they get axed? In clinics. They say, no we can't use that. To medical staff, some of them are just incredibly stupid. The original idea is like, okay this will really hit it big, or yeah, this will make monitoring really easy, etc and great. But in the next step, the implementation step? that's when things don't work as they are designed.
		Novelties	I found the whole sleep tracking thing sensational! For the first 2~3 weeks, I'd check the how the leaves are growing, showing it off to my friends.
	Interactivity		Like my daily routine? I want it to recognize certain patterns, and then alert me at the right times. Like, wake up, when I'm changing, or when I'm leaving the house, make the thing say "It's time to put on the device!" or something. Or, when I'm eating, say "Time to log your food!". If it hasn't been worn for awhile, maybe set another alarm for that, like, reminding me to put it on.
	Mobility		App is so much easier for data entry, and it's easily accessible, because I'm always carrying it. But I don't carry my computer with me all the time. I think that alone would make a huge difference.
	Perceived ease of use	Automation	When I'm keeping the food journal myself, I was never sure exactly how much I ate. But the website automates that process, like bread, such-and-such calories, or running for x minutes? that's this much calories, all based on my speed and what not, so it felt like I had my own personal trainer.
		Convenience	I think as long as it's easy to use, it'll be helpful. Convenience is really important.
		Fun	A friend of mine really thought it was annoying. But to me, it wasn't annoying, but really fun. So I kept it up almost daily, wrote down everything. My mom was like, wow that's cool, and showed interest in it too.

Perceived usefulness	Effectiveness	For me, the pedometer functionality was really nice, I'd set a goal. When I enter things, the website shows me things like, "Walk 10,000 steps today". Then I get motivated and I'd just walk around aimlessly, or stand up. At first I was really pumped. Even if I could've just gotten on the campus shuttle, but I'd walk instead, looking at the numbers. When I get 10,000 steps I'd be really proud..
	Functional usefulness	I liked the smartphone app, and it was helpful. It didn't need me to go sit in front of a computer, and let me just enter things right away. If I do it at night, I have to enter the whole day, and have to remember everything. But if I eat cookies, right away, I can just look at the wrapper, but if not, I don't know how much to enter. So if I do it right away, it's easier, or maybe like a barcode scan would be really nice, or QR code.
	Health management	For young people, they are obviously into the whole diet stuff, but for older people, activity level and food management might not come across as that difficult. I think this is for people who doesn't have any serious diseases...
Performance	Guideline	I didn't realize that the website had such functionalities, I just wrote down everything myself.
	Multipurpose	For example, MP3 players or smartphones are really fun, and we tend to focus on it. And we know that if our phone is out of reach for just a little bit, we know right away. I think instead of being a stand-alone device, it would be nicer to have it be a part of multifunctional device. That way we can always remember to bring it with us.
	Self-tracking	The really important factor is self-tracking. Before, I didn't really have a clear idea of what I was like. But after we quantify it, I can clearly see it, and think, ah this is how I am. Like, being able to reflect. I think that's one of the really crucial function of this thing.
Reliability	Inferior goods	At first I did it well.. but the little metal prongs when it got damaged.... I knew last week, Monday 15,000, the week before that, 15,000. But this week, it says 6,000? And I'm like, oh the device is faulty, and lost interest.
	Operational error	This thing sometimes stops tracking. I'd be exercising, and then look at it to check, but at some point it error'd and it didn't track what I did. Sometimes I'd be curious as to when the numbers go out, so I'd just sit really still and just move my arms, and the count went up... so I couldn't really trust it...
Scalability		I think for patients that are admitted, and has anxiety, can't sleep, those things don't get recorded. So maybe this device can connect directly to EMR, and leave a record of how much the patient had slept. I think that'd be nice.

	Visibility	I would sometimes wear it on my wrist, and since everyone can see it, people are like, wow what's that?? They'd ask a lot of questions. When I explain, they're all like, wow, I want one too. I heard that a lot.
Personal factors	Habitualization	Once this becomes a habit, I don't have to make such conscious effort to use it. But the real challenge is making the habit stick. If I had used it for longer time and tried to make the habit stick, I think I would've kept it up longer... maybe...
	Motivation	For annual member paying fees, it should send you messages like, congratulations! or some sort of motivation so that I'd keep going. If it's always the same, people lose interest. I think that's really crucial.
	Regularity of life	Daily routine is pretty set during the week, sitting in front of computer, and what not. I think the pattern really matters, regular or irregular.
	Self-reflection	Before, I didn't have any idea what I was doing, but when I see it quantified, I'd think back and go, oh so that's how I am. So that's one thing that's hard to ignore.
	Sensitiveness	It's my own thing, but I have a problem falling asleep. And when I put this on, it became even harder for me to fall asleep because I'm so aware of it. Since I know that it recognizes my movement, and I really want to sleep, but I'm tossing and turning, all the while trying to hold my arm steady.
Social factors	Competition	I think it's really important to get together and talk about it. When I was doing the study, I had three friends in the participation group - we definitely motivated each other and as competitive. Who had the biggest bloom or something little like that. "I have the biggest flower!" someone would say. On the days that I forgot to bring it, I'd be a little sad, because all my friends are doing it.
	Cultural difference	The food lists have things like greasy pizza or chocolate donut that Korean people don't really eat much. And we have different food here,... so culture difference would make it hard to bring it here.
	Generation gap	For younger people, all of this is really easy, but they don't really care about health or disease. On the contrary, older generation has a lot of motivation, but they have lower acceptability for technology. So there needs to be something to be done about that.
	Life cycle	During school days, I have set cycle - exercise in the morning, eating and everything. So it's easy to enter all that data. But after the finals, I come home for the break or some other events come up. Then, I would indulge myself with some foods, an it cuts down on my motivation..
	Recommendation	My dad just got diagnosed with high blood pressure. So, I thought it might be really good for him to get one of these to increase activity level. But, if I tell him that you need to use the Internet and manage your diet, I feel like he would be less likely to use it. But, even if he tracked just the walking distance daily, I think that would be helpful. This device, it's prettier than a regular pedometer, it's got flowers and fun to look at. Maybe that would give my dad some motivation, I thought. Young kids are interested in dieting so it would work, but maybe older people would be receptive to that too?

Attitude	Fear of envisage		Sometimes, even if I ate something, I'd be like, oh that's not that bad and decided not to log it in the journal. Or if there was a day that I ate a lot, I dreaded logging. I want to just say I didn't eat that, and really didn't log them. Like, I am okay with exercises, but foods, I want to forget about it..
Behavioral intention	Nonusage	Abandonment	If I start the day with a good log, I want to keep that all throughout the day. But if I forget it in the morning or didn't wear the device, I feel like the whole day is ruined, so I just give up for the day..
		Cost	When I was told that the price was $99, we were all like, ohhh... that's where we felt disconnected. Most of my friends and kids my age can't afford that.
		Forgetfulness	Some people forget that it's in their clothes, and put it in their laundry!! Once you forget, you don't think about it. Sometimes I leave home without thinking, and when I come home to see the device, I'm like, oh shoot... why didn't I wear it. I think maybe the device is too small, like I don't even know it's there.
		Inconvenience	App and mobile sites have significant differences, more than you think. You get your phone, go to the Internet, enter the fitbit website, log in, etc. And even when you're there, it's not easy to use, because the screen is too small, if the website isn't optimized for viewing, it's hard to use it... it was most challenging for me to get to a computer and manually enter everything. I do use other apps from the Web, but they're much easier to use. Even though they lack functionalities compared to fitbit, I tended to gravitate towards using those.
		Language barrier	Having to use an English product, that was challenging for me. Spending time and everything, I can get over that, but English is such a huge problem to overcome. And all the menu is designed for Americans, so it made me want to use it less
		Life pattern change	Around exams, I would stay up all night, and so I'd forget to charge it. And then I can't use it, and then that's probably when I started to stop keeping track. Around then..
		Lost	I heard somebody lost it the first day. They wore it like this, and when they were running because they were late for a class, it just fell out or something. I heard about someone who lost it when they wore it on their belt.
		Lost willpower	When I have the will power, I'd manually enter my sleep time and what not, but when I lose it? It's so hard to keep it up. I don't want to get on a computer and log on and enter all this information.
		Low priority	Around exam times, doing this takes the lowest priority. That's why it never got done, I can't concentrate. Exam takes my top priority. If I had some down time, I might be able to write things down, but if not? everything takes a backseat. And once you stop entering the data, it's really hard to come back and start up again.
		Reliance	I think I kept it up for about two and a half months, but when I started seeing device errors, I lost interest. I just didn't feel like doing it... and if it doesn't keep accurate track of things, I didn't feel the need to keep using it.
		Seriousness	I don't think this is all that helpful for actually sick people. This is for relatively health people trying to improve their health. So if you gave this to someone who's sick right now, and say, would you like to use this?

		Sustainability	At first, I'm all motivated, telling people how good it is, but after a while, I wasn't setting a great example...
		Uselessness	Some of the things really have no special features. BMI measurement, I can just calculate that myself. So I felt like it didn't have much to offer, and stopped using it.
	Usage	Beautification	The times where I really feel like I should go on a diet is when I want to wear clothes without being self-conscious. I want to be able to wear pretty clothes...
		Feedback	When I walk 5000 steps, it would tell you, "You've walked 500 steps today". Because of that, the next time, I'd be like, okay next time 10,000 steps, or 15,000 steps. So it felt like, I had this goal. If this was more increased, it'd be almost like having a personal trainer... It's really possible.
		Health management	I thought since I'm doing this, I will diet, manage my health, like exercise and food everything together.
		Self-satisfaction	I'd be like, wow I'm really getting fit. When I see I'm starting to get muscles, I was proud. At first, I just started to lose weight, but now I'm more serious about it an feel really satisfied. If I keep it up, I can tell that I'm doing really well and stuff...
Behavior	Arbitrary recording		Since I have to record everything, I reduced the number of items. I usually wrote it down somewhere and then entered into the website later. And sometimes even if I wrote it down, I wouldn't enter it in. I'd enter only the main items, or snacks just get left out. Sometimes I'd just increase the serving size of another food item.
	Cheating		It's easy to log on the Internet, but the effect is somewhat less significant. Since I'm not face-to-face with someone, I can just say that I'm doing well. It's not like I have evil intentions, but I was just embarrassed...
	Resolution and disconnection		When I first got it, I was like, yeah I can do this! But during the midterms, even up till the test day I was really good. But once the exams began, I just couldn't focus on it. I'd just keep thinking, okay I should do this, I should do this, and then...
	Self-monitoring		In the morning, I'm so busy, I am just out the door. After that, it's hard to go back for it. If it just stopped there, it'd be fine, but since there's no record, I lose all motivation. I just get on the bus, thinking, nobody is monitoring me today. And end up giving myself free pass to everything. I'd just take a cab, instead of walking, so there definitely was some repercussions.
	Trick		After a little bit, I only ate things with nutritional information (group laughs) or just eat the same thing over and over again. Sweet potatoes, sweet potatoes, sweet potatoes... (group laughs) because I got lazy. If you go to the school cafeteria, the hospital food actually has the calories written on it. Even if it wasn't anything really specific, at least you have something. So I'd eat the hospital food instead of the regular menu, something like that? (group laughs)
